# Nanoporous Films and Nanostructure Arrays Created by Selective Dissolution of Water‐Soluble Materials

**DOI:** 10.1002/advs.201800851

**Published:** 2018-09-13

**Authors:** Yoon Seo Kim, Jaejung Song, Chihyun Hwang, Xuejing Wang, Haiyan Wang, Judith L. MacManus‐Driscoll, Hyun‐Kon Song, Seungho Cho

**Affiliations:** ^1^ School of Materials Science and Engineering Ulsan National Institute of Science and Technology (UNIST) Ulsan 44919 Republic of Korea; ^2^ School of Energy and Chemical Engineering Ulsan National Institute of Science and Technology (UNIST) Ulsan 44919 Republic of Korea; ^3^ School of Materials Engineering Purdue University West Lafayette IN 47907 USA; ^4^ Department of Materials Science and Metallurgy University of Cambridge 27 Charles Babbage Road Cambridge CB3 0FS UK

**Keywords:** nanocomposites, nanoporous materials, nanostructure arrays, water‐soluble materials

## Abstract

Highly porous thin films and nanostructure arrays are created by a simple process of selective dissolution of a water‐soluble material, Sr_3_Al_2_O_6_. Heteroepitaxial nanocomposite films with self‐separated phases of a target material and Sr_3_Al_2_O_6_ are first prepared by physical vapor deposition. NiO, ZnO, and Ni_1−_ *_x_*Mg*_x_*O are used as the target materials. Only the Sr_3_Al_2_O_6_ phase in each nanocomposite film is selectively dissolved by dipping the film in water for 30 s at room temperature. This gentle and fast method minimizes damage to the remaining target materials and side reactions that can generate impurity phases. The morphologies and dimensions of the pores and nanostructures are controlled by the relative wettability of the separated phases on the growth substrates. The supercapacitor properties of the porous NiO films are enhanced compared to plain NiO films. The method can also be used to prepare porous films or nanostructure arrays of other oxides, metals, chalcogenides, and nitrides, as well as films or nanostructures with single‐crystalline, polycrystalline, or amorphous nature.

## 1 Introduction

Preparing films with high specific surface area is a subject that has been studied by research groups all over the world because such materials are useful in a variety of applications such as catalysis, sensing, energy harvesting and storage, gas storage, charge storage (e.g., in supercapacitors), and in adsorption. Various strategies have been exploited to prepare films with high specific surface areas, such as attachment of nanopowders on substrates, direct growth of 1D or 2D nanostructures on substrates (bottom‐up methods),^1^ anodization of metal films or substrates,^2^ selective etching of one component of a composite on a substrate by using a solution,^3^ metal‐assisted etching,^4^ and using soft templates to obtain inverse opal structures.^5^

Recently, we reported a new approach to obtain porous films with high specific surface area.^6^ Heteroepitaxial nanocomposite films composed of strontium titanate, SrTiO_3_ (STO), and magnesium oxide (MgO) with nanoscale phase separation were grown by physical vapor deposition, after which the nanoscale MgO phase was chemically and selectively etched away by an acidic solution, thus generating mesoporous STO films.^6^ These films possessed vertically aligned 1D pores with morphologies similar to those of films prepared by anodization of metal films or metal substrates.^2^, ^7^ The pore widths of the films prepared by selective etching of the heteroepitaxial nanocomposite films were much smaller (i.e., much higher specific surface areas of the films) than those of films prepared by anodization. Another striking difference between the porous films prepared by the two methods is that the films made by selective etching of heteroepitaxial nanocomposite films have quasi‐single‐crystalline nature, which can be beneficial for a range of applications that require efficient charge‐carrier transport, such as energy‐harvesting and electronic devices, because of their much smaller number of crystalline grain boundaries and epitaxial connection to the underlying materials. Although it is relatively easy to obtain porous tertiary oxides such as perovskite and spinel materials using such methods, obtaining porous binary oxides like MgO and ZnO would be more difficult because they could hardly survive in the selective etching processes generally used.[[qv: 3a,6]] Therefore, only a very limited range of porous films with a narrow range of compositions have been prepared so far by etching one phase of a composite.

In this study, we introduced a method to create porous epitaxial films or nanostructure arrays by selective dissolution of a water‐soluble component. After physical vapor deposition of a nanocomposite film with two nanoscale phases that are separated by self‐assembly, one of which is water‐soluble, the film only needs to be dipped in water at room temperature for 30 s for selective and complete dissolution of the water‐soluble phase to form a porous film or nanostructure arrays of the remaining target material. This gentle and short process uses pure water, which is the most abundant and accessible solvent for selective phase dissolution, and thus minimizes damage to the remaining target material and the chance of incorporating by‐product impurity phases produced by chemical reactions with the target material.^6^ Furthermore, the remaining film's morphology can be also tailored by controlling the relative wettability of the film on the underlying material. Strontium aluminate (Sr_3_Al_2_O_6_, SAO; space group $Pa\bar{3}$) was used as a water‐soluble material (also referred as water‐soluble phase)^8^ in our study. In addition to being water‐soluble, SAO has the benefit that its crystal lattice is well‐matched to STO, which is the most promising oxide buffer on silicon, the key end‐goal substrate in the field of electronics.^9^ A good match between the crystal structure of a film and that of the underlying material is a prerequisite for epitaxial growth. Thus, use of SAO as one phase of a nanocomposite film on an STO substrate enables epitaxial growth of each phase, which is guaranteed to have a quasi‐single‐crystalline nature (i.e., with only very low angle (<≈1°) columnar grain boundaries). By using this gentle and fast method, we were able to obtain porous epitaxial binary oxide films, porous binary oxide nanostructure arrays, and porous epitaxial solid‐solution films.

To create porous films and nanostructure arrays, for the following reasons, we chose NiO, Ni_0.5_Mg_0.5_O, and ZnO as the model target materials to be combined with the SAO sacrificial phase:

– First, NiO (space group: $Fm\bar{3}m$) is a p‐type binary oxide and a promising material for electrochemical applications such as supercapacitors, electrocatalysts, and electrochemical sensors.^10^ High crystalline quality, high surface area NiO films would have significant advantages over standard nanoporous polycrystalline materials, or plain films.
– Second, by demonstrating the formation of Ni_0.5_Mg_0.5_O (same structure as NiO and MgO, space group: $Fm\bar{3}m$) as a porous “solid‐solution” film, this would demonstrate the broad applicability of our method for creating a wide range of nanoporous materials properties.
– Third, hexagonal wurtzite ZnO (space group: *P*6_3_ *mc*) is a very promising material in a wide range of applications.^11^ It has been widely used as a sacrificial material in selective‐etching processes.[[qv: 3a-c]] However, it is also very important in nanowire form for applications in optoelectronics.[[qv: 1a,12]] Hence, successful production of ZnO as a target material giving 1D ZnO nanostructure arrays, when it is normally the easily etched material, would be an indication of the generality of our method. For our nanocomposite films, unless the volume fraction of the ZnO phase in the film is well in excess of 50%, the poor crystallographic compatibility of ZnO (hexagonal) on the STO (cubic perovskite) substrate would lead to poor wettability. On the other hand, the similar lattice matching of SAO means it has good wettability to the STO substrate. Hence, the SAO would form a matrix phase and the ZnO would be expected form as nanopillars within it. After selective dissolution of the SAO matrix, one would therefore expect to form substrate‐supported, freestanding ZnO nanopillar (nanowire) arrays. The crystal systems and lattice constants of the materials in this study are provided in the Supporting Information (Table S1).

## 2 Results and Discussion

**Figure 1:**
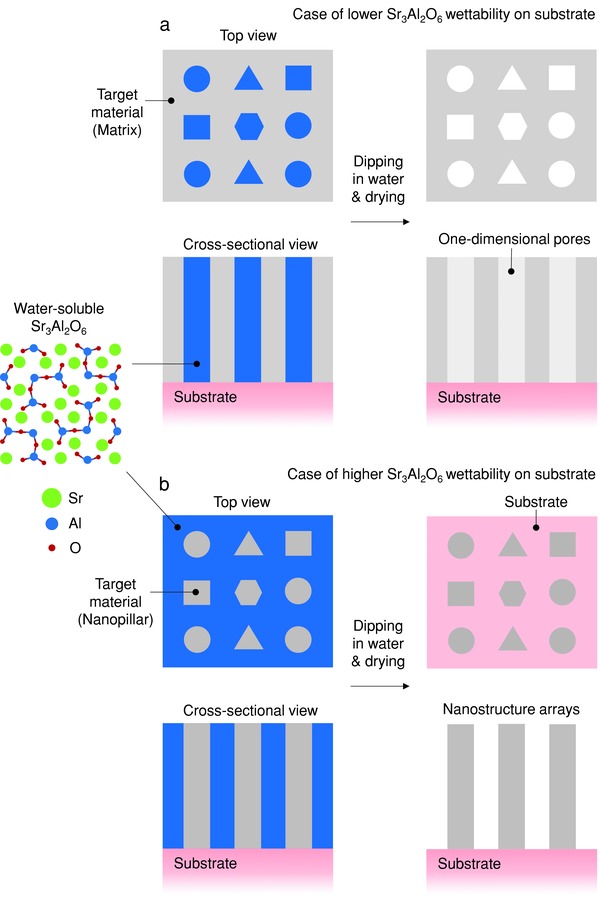
Schematic diagram of formation of a film with 1D pores or 1D nanostructure arrays by selective dissolution of the water‐soluble Sr_3_Al_2_O_6_ phase of a nanocomposite film. After dipping the nanocomposite film in water and drying, a film with 1D pores or 1D nanostructure arrays are formed. a) Formation of film of target material with 1D pores when the wettability of Sr_3_Al_2_O_6_ is lower than that of the target material on the substrate. b) Formation of 1D nanostructure arrays of target material when the wettability of Sr_3_Al_2_O_6_ is higher than that of the target material on the substrate.

**Figure** **1** shows schematic diagrams of the nanocomposite structures in a film, before and after selective dissolution of water‐soluble SAO by dipping the film in water to form a porous film or nanostructure arrays. The different cross‐sectional shapes of the embedded nanopillars are schematic only and simple serve to demonstrate that the pillars are not necessarily circular in cross‐section, but can have a variety of shapes depending both on the lateral interfacial energies between the materials in the film and the substrate and on the vertical interfacial energies between the two materials in the film.^13^

If SAO has lower wettability than the target material on the substrate, if the two phases are immiscible, and if their volume ratios are not significantly different, then the target material would be expected to form the matrix, and the SAO phase nanopillars would form within this matrix. Pores would be expected to form after the nanopillars are selectively etched out of the film (Figure 1a).

In the opposite case with respect to the relative wettabilities of SAO and the target material, an SAO matrix and nanopillars of the target material will form in the nanocomposite film. After the nanocomposite film is dipped into water and dried, nanostructure arrays of the target material are obtained on the substrate (Figure 1b). It should also be noted that when the wettabilities of both phases on the substrate are relatively low, then less ordered structures compared than shown in Figure 1a,1b might be expected to be observed.

**Figure 2:**
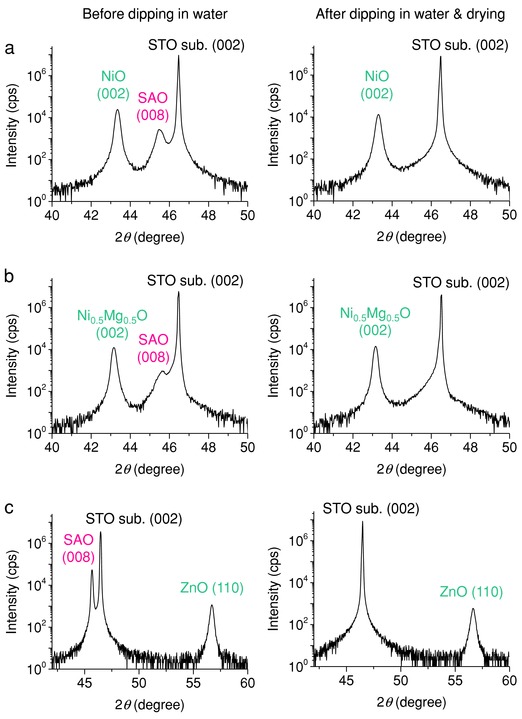
X‐ray diffraction (XRD) ω–2θ scans. XRD ω–2θ scans of nanocomposite films grown on SrTiO_3_(001) substrates before and after dipping in water at room temperature for 30 s and drying (cps: counts per second): a) NiO–Sr_3_Al_2_O_6_ nanocomposite film; b) Ni_0.5_Mg_0.5_O–Sr_3_Al_2_O_6_ nanocomposite film; c) ZnO–Sr_3_Al_2_O_6_ nanocomposite film.

In our study, self‐assembled nanocomposite films (NiO–SAO, Ni_0.5_Mg_0.5_O–SAO, and ZnO–SAO) were first deposited on STO substrates by pulsed laser deposition (PLD). The X‐ray diffraction (XRD) ω–2θ scans of the NiO–SAO, Ni_0.5_Mg_0.5_O–SAO, and ZnO–SAO nanocomposite films show only (00*l*) peaks of SAO, NiO, and Ni_0.5_Mg_0.5_O, and the (110) peak of ZnO, and hence all three compositions show excellent phase separation and a high degree of crystallographic orientation (see XRD data before the films were dipped into water, shown in **Figure** **2** and Figure S1 in the Supporting Information). After the films were dipped into water for 30 s and dried, only the SAO peak in the XRD patterns disappeared, indicating, in all three cases, selective dissolution of the SAO phase (see XRD data after water dipping, shown in Figure 2 and Figure S1 in the Supporting Information). The ϕ‐scans around STO(202) and NiO(404) of the NiO–SAO film after water dipping display a set of four peaks at the same ϕ angles, indicating the epitaxial relationship between the film and STO substrate (Figure S2 in the Supporting Information).

**Figure 3:**
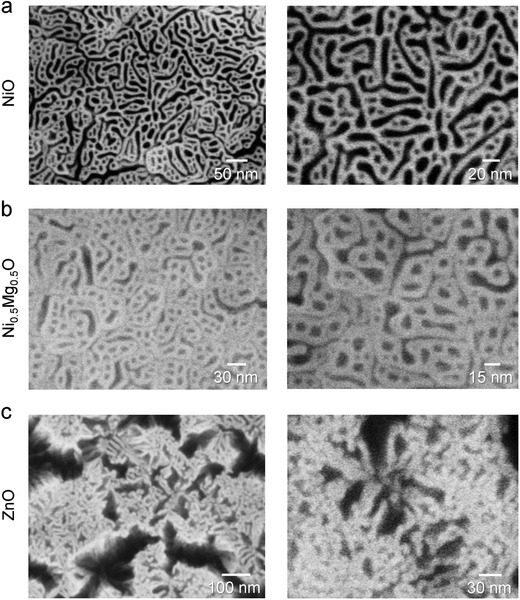
Scanning electron microscope (SEM) images of porous films and nanostructure arrays after selective dissolution of the water‐soluble phase. Top‐view SEM images of resulting porous films after dipping nanocomposite films grown on SrTiO_3_(001) substrates in water and drying: a) NiO–Sr_3_Al_2_O_6_ nanocomposite film; b) Ni_0.5_Mg_0.5_O–Sr_3_Al_2_O_6_ nanocomposite film; c) ZnO–Sr_3_Al_2_O_6_ nanocomposite film.

**Figure** **3** shows top‐view scanning electron microscope (SEM) images of the films after they were dipped in water and after the wet films were dried. For the NiO–SAO nanocomposite film the SEM images indicate that the matrix was NiO and the isolated phases were SAO before water dipping. When the nanocomposite film was dipped into water, the SAO phase was selectively dissolved to form the porous NiO film (Figure 3a). In the case of the Ni_0.5_Mg_0.5_O–SAO nanocomposite film, after selective dissolution of SAO, a similar porous Ni_0.5_Mg_0.5_O film (Figure 3b) formed on the STO. Hence, both porous solid‐solution pure films and solid‐solution films are readily formed, with the film composition controlled simply by varying the PLD target composition.

Figure 3c shows the ZnO–SAO nanocomposite film after water immersion showing this film to be porous. The tilt‐view images (Figure S3 in the Supporting Information) reveal that arrays of ZnO nanowires with an average diameter of ≈10 nm formed after the film was dipped into water, indicating that the SAO matrix surrounded the ZnO nanopillars before immersion, as already predicted based on the poorer wettability of ZnO on STO cf. SAO on STO. Consequently, the SAO matrix was dissolved by water so that ZnO nanopillars were exposed, as shown in Figure 3c and Figure S3 (Supporting Information). One inadvertently broken nanowire is shown in Figure S3b (Supporting Information). ZnO nanowires coalesced into bundles after SAO dissolution as clearly seen in the top‐view image (Figure 3c), tilt‐view image (Figure S3 in the Supporting Information), and low‐magnification top‐view images (Figure S4 in the Supporting Information). The exposed and aligned ZnO nanopillars (nanowires) were forced to bend and be drawn together by surface tension forces induced by the evaporation of water between the nanowires in the drying process.^14^

To further explore the importance of the relative substrate wettabilities of the two phases in the film, we deposited a ZnO–SAO nanocomposite film on a ZnO(001) substrate using the same film‐growth conditions as those for the growth of the ZnO–SAO nanocomposite film on the STO substrate. The XRD patterns of the nanocomposite film grown on the ZnO(001) substrate before and after water immersion are provided in Figure S5 (Supporting Information). After water dipping and drying, the SAO(044) peak disappeared from the XRD pattern. Figure S6 (Supporting Information) shows top‐view SEM images of the ZnO–SAO nanocomposite film grown on the ZnO(001) substrate after selective dissolution of SAO. In contrast to the ZnO–SAO nanocomposite film deposited on the STO substrate (Figure 3c), where SAO forms the matrix phase, here for growth on ZnO, ZnO forms the matrix phase (Figure S6 in the Supporting Information). We observed a porous structure formed in a ZnO matrix, rather than ZnO nanowires as demonstrated for the growth on STO. Hence, the porous film morphology is completely and simply changed by controlling the relative substrate wettabilities of the two phases in the film, i.e., when the substrate is changed, the film morphology is changed markedly from nanowires to porous matrix.

**Figure 4:**
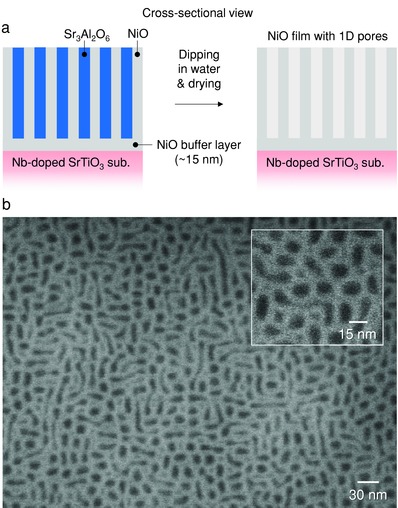
Control of relative wettability by using buffer layer. a) Schematic diagram of formation of film with 1D pores on buffer layer by selective dissolution of the water‐soluble phase. b) Top‐view SEM images of porous NiO film on NiO buffer layer after selective dissolution of the water‐soluble Sr_3_Al_2_O_6_ phase.

The strong dependence of film microstructure on substrate wettability was also explored for the NiO/SAO system. We use a NiO buffer layer on Nb‐doped STO(001) before growth of a NiO–SAO nanocomposite film. The NiO buffer layer was ≈15 nm thick, as illustrated in **Figure** **4**a. The NiO–SAO layer was grown on the NiO without breaking vacuum by rotating a PLD target carousel to prevent contamination at the interface between the two layers. The NiO buffer layer will enable the NiO phase in the nanocomposite layer to completely wet it. As a result, the SAO phase was less elongated in the lateral (in‐plane) directions than the SAO phase in the film directly grown on STO (i.e., without the NiO buffer, Figure 3a), thus giving rise to smaller pore widths after selective dissolution of SAO, as shown in Figure 4b. We note that while it is already well known that composite film morphologies in physically vapor deposited films are controlled by kinetics parameters,^13^, ^15^ here, for the first time we demonstrated the control of nanocomposite morphology using thermodynamic parameters, namely the relative wettabilities of the different phases in the film with the underlying substrate/buffer.

**Figure 5:**
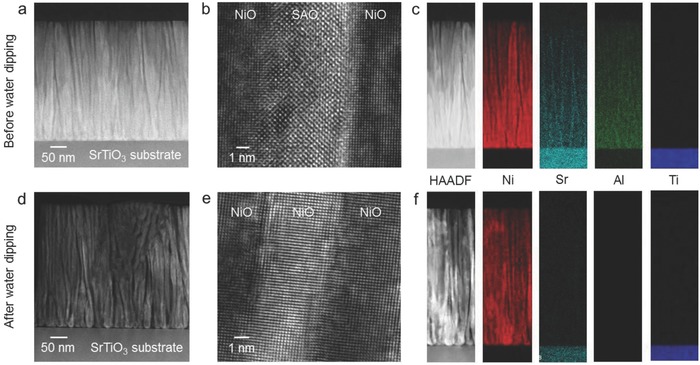
Results of scanning transmission electron microscopy (STEM) of NiO–Sr_3_Al_2_O_6_ nanocomposite films grown on SrTiO_3_(001) substrates. a,b) Cross‐sectional STEM high‐angle annular dark‐field (HAADF) images of as‐grown film. c) Cross‐sectional HAADF image and corresponding energy‐dispersive X‐ray spectroscopy (EDS) elemental maps of as‐grown film. d,e) Cross‐sectional STEM HAADF images after water dipping and drying the film. f) Cross‐sectional HAADF image and corresponding EDS elemental maps after water dipping and drying the film.

Since the SEM images of Figure 3 do not show through thickness information to determine whether the SAO (and only SAO) was fully etched out of the films after water dipping, scanning transmission electron microscopy (STEM) was performed to determine this. **Figure** **5**a shows a cross‐sectional STEM high‐angle annular dark‐field (HAADF) image of the NiO–SAO nanocomposite film. Nanoscale phase separation was observed,^13^ and each phase was connected from the bottom to the top of the film. The NiO and SAO phases had distinct crystal structures, with an epitaxial relationship between the two phases (Figure 5b).

**Figure 6:**
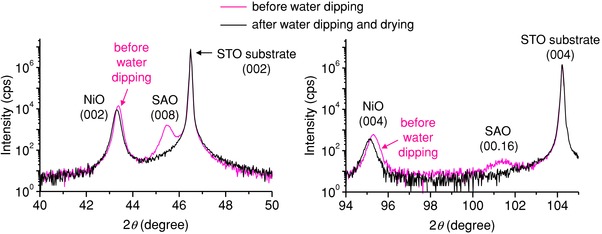
XRD ω–2θ scans of NiO‐Sr_3_Al_2_O_6_ (SAO) nanocomposite film grown on SrTiO_3_ (STO)(001) substrate before and after dipping in water and drying.

For further proof and understanding of the vertical epitaxial connection between the phases in the film, we explored the strain in the two materials using X‐ray diffraction. The *a* (or *c*) lattice constants of bulk SAO and bulk NiO are 1.5856 nm (ICSD 98‐007‐1860) and 0.4176 nm (ICSD 98‐002‐8910), respectively (Table S1 in the Supporting Information). If the 4*a* (or 4*c*) value of 1.6704 nm for bulk NiO and the domain epitaxy are considered,^16^ the NiO phase that was epitaxially connected with the SAO phase is expected to accommodate the compressive vertical (i.e., along the *c*‐axis direction) strain in a NiO–SAO heteroepitaxial nanocomposite film. As shown in the XRD patterns (**Figure** **6**) of the NiO–SAO nanocomposite film before and after water dipping and drying, the NiO(002) and NiO(004) peaks shifted toward lower 2θ values after dissolution of the SAO phase. This indicates that before water dipping, the NiO phase was compressively strained along the *c*‐axis direction, and the out‐of‐plane cell constants of the NiO phase increased (from 0.4170 to 0.4176 nm) after water dipping because of vertical‐strain relaxation arising from dissolution of the epitaxially connected SAO phase. Despite the relaxation of the compressive strain in the NiO after dissolution of the SAO, the phase remained very stable and there was no crack formation with release of the strain.

From the STEM observations, we found that the interfaces were very clean with no interphases. The chemical composition of the film was investigated by energy‐dispersive X‐ray spectroscopy (EDS). The EDS elemental maps (Figure 5c) confirmed the separation of the NiO and SAO phases in the film. The portion of the HAADF image that showed the film dipped into water and dried was darker than the portion of the image that showed the film before water dipping (i.e., comparing Figure 5a,d). The STO substrates showed the same brightness (Figure 5a,d). The difference in brightness in the film region could be induced by mass reduction of the film as a result of selective dissolution of SAO by water. The high‐resolution HAADF image (Figure 5e) showed that the brighter part and the darker parts correspond to portions of the film with the same crystal structures, indicating that the contrasts in Figure 5d,e do not originate from elements with different atomic numbers, but from the spatial mass difference [i.e., variations in thickness of the film in the cross‐sectional TEM specimen, where the parts with pores were thinner (darker)]. The EDS elemental maps of the film after water dipping and drying confirm that selective dissolution of SAO took place, leaving only the NiO phase on the substrate (Figure 5f).

**Figure 7:**
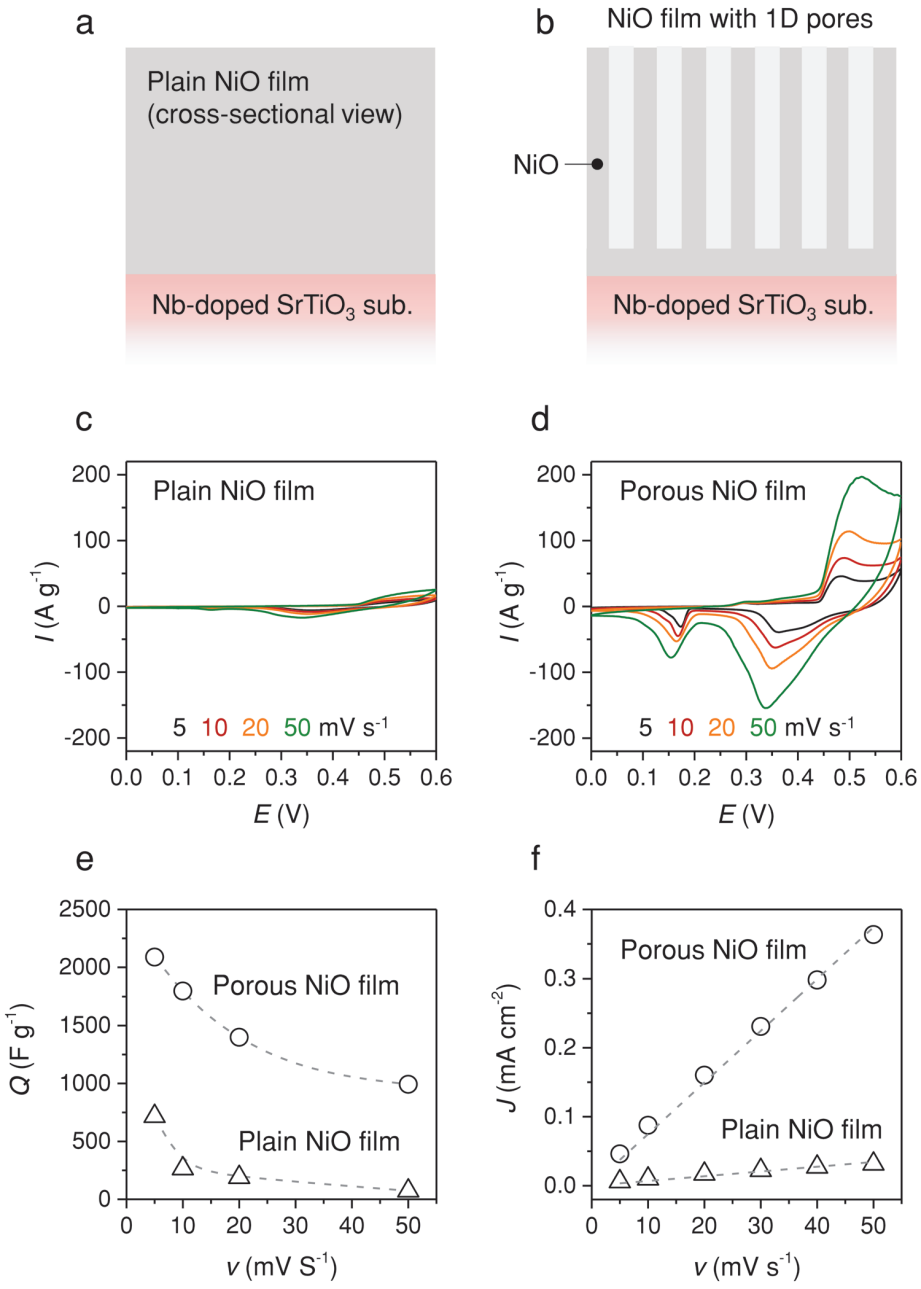
Electrochemical behavior of plain and porous NiO electrodes. a,b) Cross‐sectional schematic illustrations of plain and porous NiO electrodes. c,d) Cyclic voltammograms obtained at scan rates of 5, 10, 20, and 50 mV s^−1^. e) Variations in specific capacitance with scan rate for both electrodes. f) Variations in double‐layer charging current at +0.05 V versus scan rate.

The supercapacitor properties of a porous NiO film grown from the NiO–SAO nanocomposite film on the NiO‐buffered Nb‐doped STO(001) substrate were then determined. NiO is well known as an active electrode materials for electrochemical supercapacitors and the theoretical specific capacitance is 3750 F g^−l^.[[qv: 10a,b,17]] After selective dissolution of SAO, the NiO buffer layer prevented exposure of the substrate to the solutions used in the electrochemical measurements. The electrochemical properties of a control, plain NiO film on a Nb‐doped STO(001) substrate were also measured (**Figure** **7**a,b). A cyclic voltammetry study was conducted on the porous and plain NiO film electrodes, using 1.0 m KOH as an electrolyte with a potential window of 0 to 0.6 V (vs Hg/HgO), at different scan rates in the range of 5–50 mV s^−1^ (Figure 7c,d). The relative specific capacitance decreased with increasing scan rate. The calculated values of the specific capacitance were 2088 F g^−1^ at the scan rate of 5 mV s^−1^ for the porous NiO electrode, which is higher than those of the plain NiO electrode (716 F g^−1^) and reported porous NiO films and NiO nanostructure arrays.^18^ High specific capacitance values of 1797, 1398, and 993 F g^−1^ at the scan rates of 10, 20, and 50 mV s^−1^, respectively, were obtained for the porous NiO electrode (Figure 7e). These values are also higher than those of the plain NiO electrode (265, 189, and 73 F g^−1^ at the scan rates of 10, 20, and 50 mV s^−1^, respectively). Quantitative analysis of the electrochemically active surface area (ECSA) on the electrodes was also conducted.^19^ Figure S7 (Supporting Information) shows the cyclic voltammograms of both electrodes obtained at different scan rates (5–50 mV s^−1^) in the potential range of 0–0.1 V, where no Faradaic reaction occurred. Based on the results, current densities at +0.05 V were plotted against the potential scan rates (Figure 7f). The double‐layer capacitance (*C*_dl_, proportional to ECSA) of the porous NiO electrode was estimated from the slope of the plot to be 7.6 mF cm^2^, which is higher than that of the plain NiO electrode (0.7 mF cm^2^). No noticeable structural difference was observed between the porous NiO film before and after 100 cycles in the electrochemical test, indicating the high mechanical stability of the porous NiO film (Figure 4b and Figure S8 in the Supporting Information).

Compared with nanopowders, porous materials that are perfectly immobilized on the substrate prepared by this method have merits in applications which need charge‐carrier transport. Thus, while a nanopowder has the advantage over bulk counterparts of higher surface‐to‐volume ratio (i.e., higher number of reaction sites), the higher number of grain boundaries and poor particle‐to‐particle contact in a coated nanopowder interrupts charge transfer. Furthermore, the specific surface area is substantially reduced after it is coated and treated on a substrate to give powder immobilization.[[qv: 1b,20]] In this context, the epitaxial nature of the porous films and nanostructure arrays prepared by selective dissolution enables enhanced charge‐carrier transport. Hence, in addition to the elimination of charge‐blocking‐high angle grain boundaries, a porous film made by this method is also epitaxially connected to its underlying substrate, which further benefits charge transferability. Hence, the porous films and nanostructure arrays prepared by this method have strong potential for improved performance over chemically grown materials.

Another potential advantage of the gentle and short etching process nanoporous films compared to chemically grown material or selectively etched materials using acidic or basic solutions^21^ is that there will be fewer surface chemical reactions or destructive physical events taking place. Thus, lower defect concentrations or fewer surface phases as by‐products are expected. On the other hand, the PLD process used in this work for preparing the mother heteroepitaxial nanocomposite films is not scalable. In this context, epitaxial materials can be prepared nowadays by scalable, industrial crystal growth routes such as reactive co‐evaporation.^22^ A mother nanocomposite film can be prepared in a similar manner. In addition, porous films and nanostructure arrays prepared from heteroepitaxial nanocomposite films also have the potential to be integrated into state‐of‐the‐art silicon technology because epitaxial growth of plain or nanocomposite films on silicon is already an established technique.^9^

## 3 Conclusion

Porous epitaxial films or substrate‐supported nanostructure arrays were prepared by a simple procedure of dipping nanocomposite films in water for 30 s to dissolve a water‐soluble phase out of nanocomposite films. NiO, Ni_0.5_Mg_0.5_O, and ZnO were used as test binary systems. Mostly perovskite or spinel nanocomposites have been studied, although simple binaries have many advantages for applications. The morphologies of the pores and nanostructures followed the phase‐separation morphologies of the nanocomposite films before selective dissolution of the water‐soluble phase. The pore morphologies were highly tunable by controlling the morphology of the parent nanocomposite film through selection of the appropriate substrate (or buffer layer) to give either high or low film/substrate wettability. An exemplar functional property to demonstrate the strong benefit of obtaining highly nanoporous epitaxial films was the supercapacitor property of the porous NiO film, which was strongly improved compared to that of the plain film. Although NiO, Ni_0.5_Mg_0.5_O, and ZnO were chosen as the target materials in this study, this method can be extended to the preparation of films of other materials with high specific surface areas by using composite films composed of a water‐soluble material and other metal oxides, metals, metal chalcogenides, metal nitrides, etc.^23^ This method can also be utilized for a nonepitaxial system to form porous polycrystalline films and nanostructure arrays, and polycrystalline substrates can be used for the growth of nonepitaxial composite films with water‐soluble phases. Using selective dissolution of the water‐soluble phases in the nonepitaxial composites, porous polycrystalline films and nanostructure arrays can be obtained on the polycrystalline substrates. Therefore, the range of selectable materials can be significantly extended by using nonepitaxial composites.

## 4 Experimental Section

*Sample Preparation*: The films were grown on STO(001), 0.5 wt% Nb‐doped STO(001), or ZnO(001) substrates by PLD using a KrF laser (λ = 248 nm) with a fluence of 2.25 J cm^−2^ and a repetition rate of 1 Hz. A polycrystalline target containing NiO and SAO in a volume ratio of 0.5:0.5, a polycrystalline target containing ZnO and SAO in a volume ratio of 0.5:0.5, and a polycrystalline target containing Ni_0.5_Mg_0.5_O and SAO in a volume ratio of 0.5:0.5 were used to fabricate the NiO–SAO nanocomposite films, ZnO–SAO nanocomposite films, and Ni_0.5_Mg_0.5_O–SAO nanocomposite films, respectively. The target‐to‐substrate distance was 5.5 cm. During deposition, the substrate temperature was set at 730 °C, and the O_2_ pressure was fixed at 0.2 mbar. The nanocomposite films were dipped into deionized water at 20 °C for 30 s for selective dissolution of their SAO phase to obtain porous films or nanostructure arrays.

*Characterization*: The crystalline nature of the resulting films and nanostructure arrays was investigated by XRD on a high‐resolution X‐ray diffractometer (Empyrean, PANalytical, The Netherlands) using Cu K_α_ radiation (λ = 1.5405 Å). To investigate the in‐plane orientation of these films and nanostructures, ϕ scans were performed by rotating each sample by 360° in the in‐plane direction. The morphologies of the films were characterized by scanning electron microscopy (SEM; S‐4800 Hitachi, Japan). A scanning transmission electron microscope (STEM; Titan G2 80−200, FEI, USA) with a Cs probe corrector and ChemiSTEM technology (high‐brightness field‐emission gun and SuperX energy‐dispersive X‐ray spectrometer with four windowless silicon‐drift detectors), operated at 200 kV, was used to evaluate the structural properties and obtain energy‐dispersive EDS chemical mappings. Cross‐sectional samples for STEM analysis were prepared by standard manual grinding, and the samples were thinned with a final ion‐milling step using a precision ion‐polishing system (PIPS 691, Gatan, USA). The electrochemical performances of the NiO films were evaluated using a Pt foil as the counter electrode, Hg/HgO as the reference electrode, and a KOH solution (1 m) as the electrolyte. The cyclic voltammetry measurements of the NiO electrode were performed at different scan rates using a computer‐controlled electrochemical interface (VMP 3).

## Conflict of Interest

The authors declare no conflict of interest.

## Supporting information

Supplementary
