# Supplementary material for: Nanoporous Films and Nanostructure Arrays Created by Selective Dissolution of Water‐Soluble Materials
Source: Adv Sci (Weinh). 2018 Sep 13;5(11):1800851. doi: 10.1002/advs.201800851 (PMC6247061; doi:10.1002/advs.201800851)
Supplement: Supplementary file 1 — Supplementary [file ADVS-5-1800851-s001.pdf]

Copyright WILEY-VCH Verlag GmbH & Co. KGaA, 69469 Weinheim, Germany, 2018.

ADVANCED SCIENCE
Open Access

Supporting Information

for *Adv. Sci.,* DOI: 10.1002/advs.201800851

Nanoporous Films and Nanostructure Arrays Created by Selective Dissolution of Water-Soluble Materials

Yoon Seo Kim, Jaejung Song, Chihyun Hwang, Xuejing Wang, Haiyan Wang, Judith L. MacManus-Driscoll, Hyun-Kon Song,* and Seungho Cho**

Copyright WILEY-VCH Verlag GmbH & Co. KGaA, 69469 Weinheim, Germany, 2016.

Supporting Information

Nanoporous Films and Nanostructure Arrays Created by Selective Dissolution of Water-Soluble Materials

Yoon Seo Kim, Jaejung Song, Chihyun Hwang, Xuejing Wang, Haiyan Wang, Judith L. MacManus-Driscoll, Hyun-Kon Song,* and Seungho Cho**

Table S1. Crystal systems and lattice constants of model materials and substrates.

	$SrTiO_3$	$Sr_3Al_2O_6$	NiO	$Ni_{0.5}Mg_{0.5}O$	ZnO
Space group (Crystal system)	*Pm*-3*m* (Cubic)	*Pa*-3 (Cubic)	*Fm*-3*m* (Cubic)	*Fm*-3*m* (Cubic)	$P6_3mc$ (Hexagonal)
In-plane lattice constant (*a*) [Å]	3.905	15.856	4.176	4.193	3.249
Out-of-plane lattice constant (*c*) [Å]	3.905	15.856	4.176	4.193	5.206

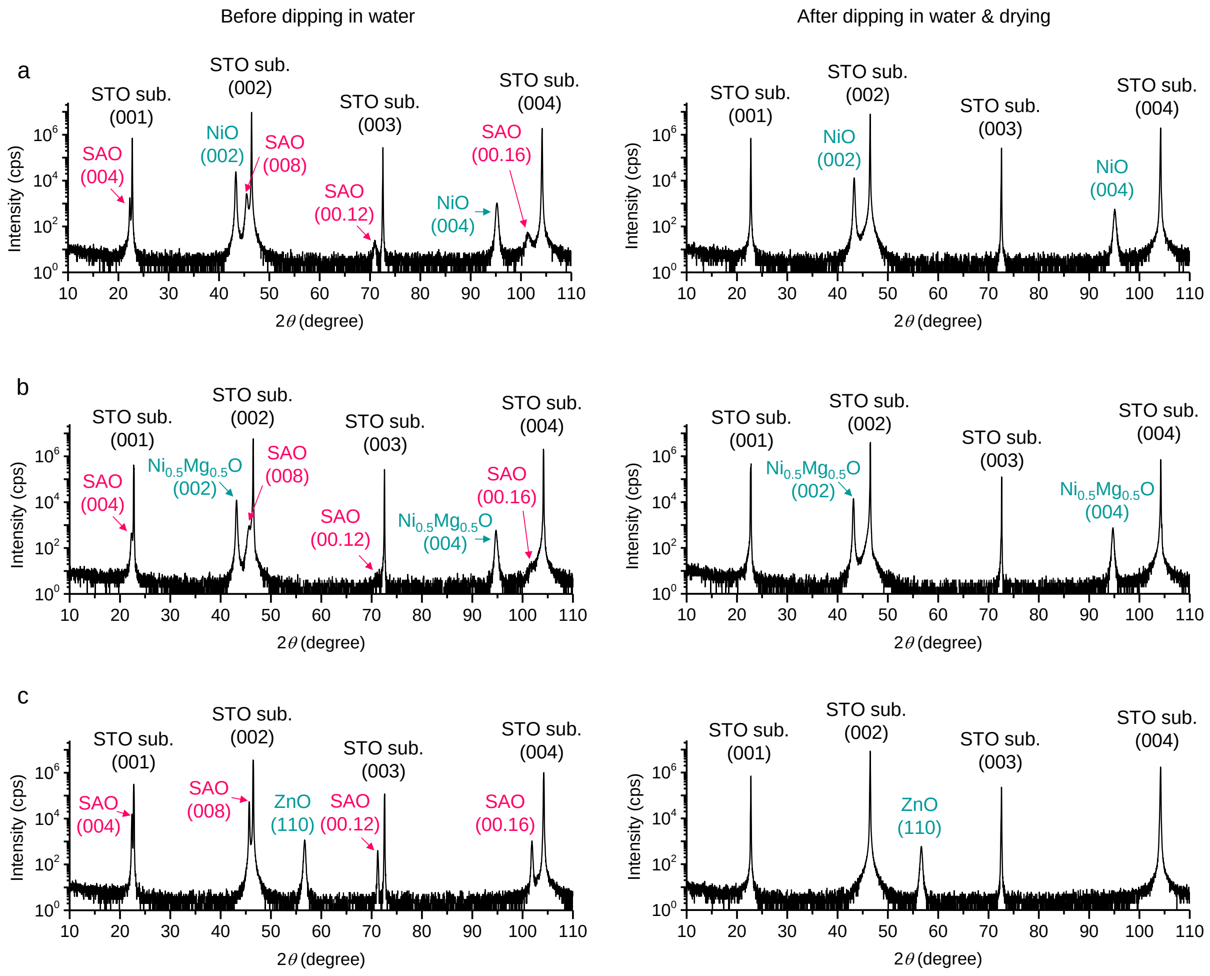

Figure S1. XRD ω-2ϑ scans of nanocomposite films grown on $SrTiO_3$ (STO)(001) substrates before and after dipping in water at room temperature for 30 s and drying. (a) NiO-$Sr_3Al_2O_6$ nanocomposite film. (b) $Ni_{0.5}Mg_{0.5}O$-$Sr_3Al_2O_6$ nanocomposite film. (c) ZnO-$Sr_3Al_2O_6$ nanocomposite film.

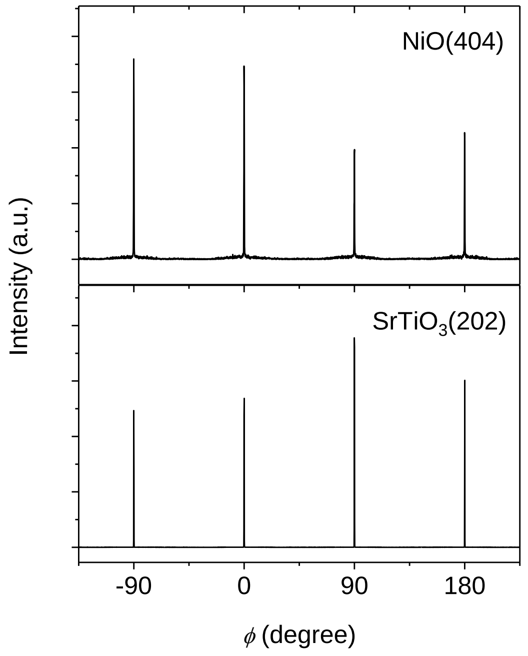

Figure S2. 360° φ-scans of $SrTiO_3$(202) and NiO(404) of the NiO-$Sr_3Al_2O_6$ film after water dipping.

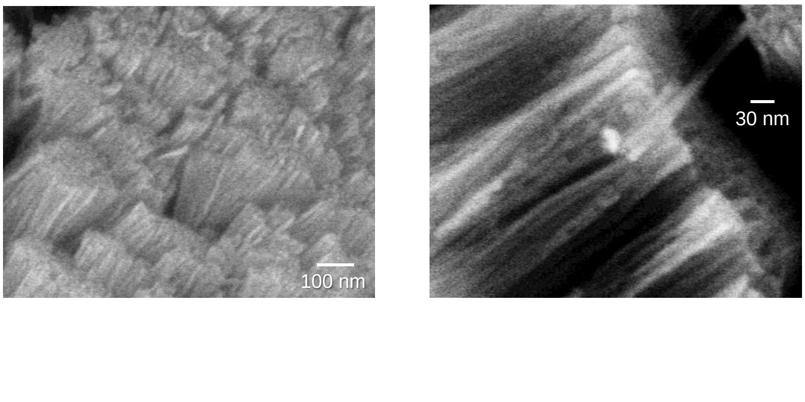

Figure S3. Tilt-view SEM images of ZnO nanostructure arrays prepared by dipping of ZnO-$Sr_3Al_2O_6$ nanocomposite film grown on $SrTiO_3$(001) substrate in water and drying.

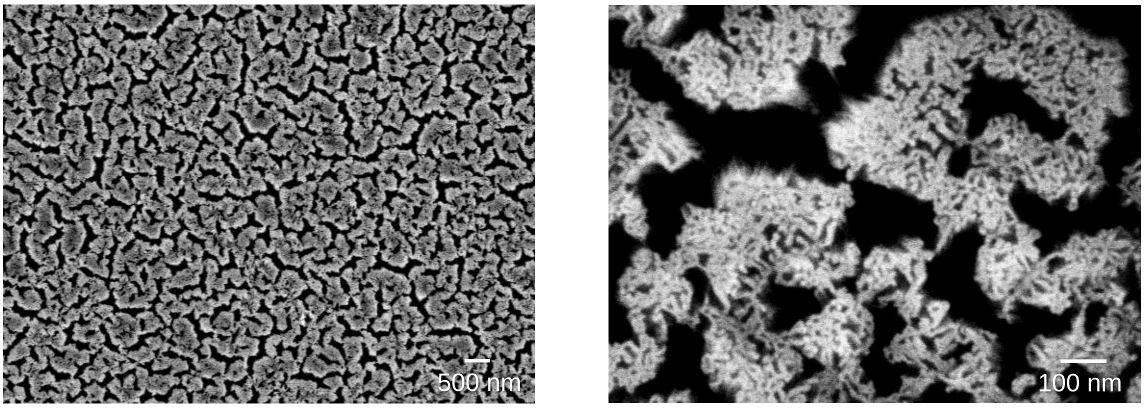

Figure S4. Low-magnification top-view SEM images of ZnO nanostructure arrays prepared by dipping of ZnO-$Sr_3Al_2O_6$ nanocomposite film grown on $SrTiO_3$(001) substrate in water and drying.

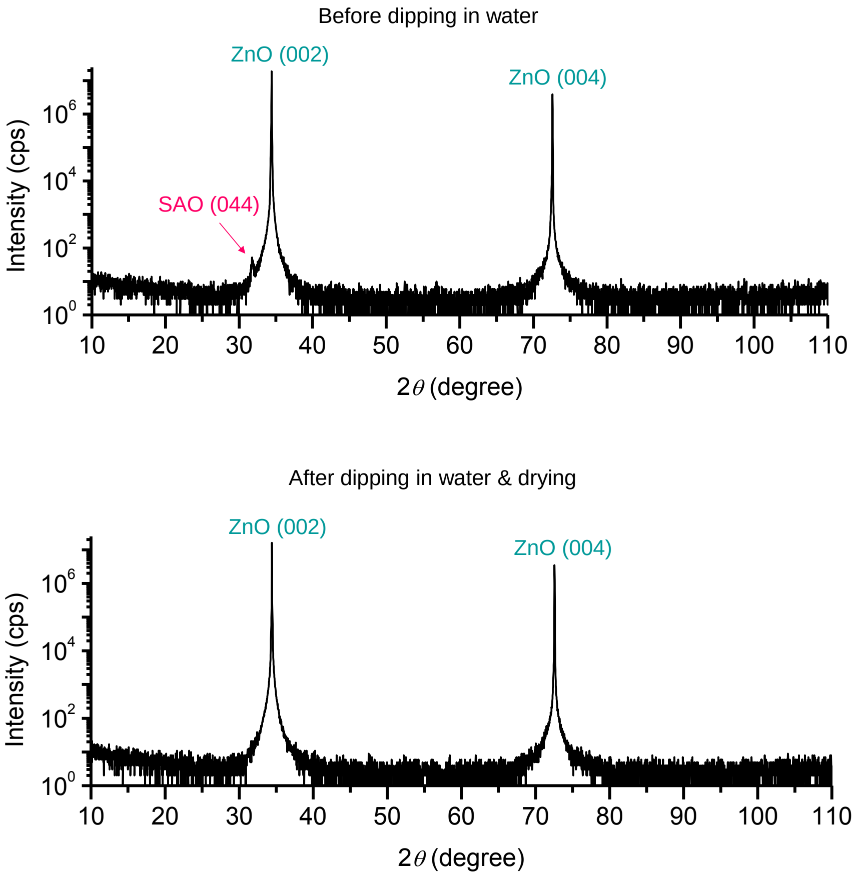

Figure S5. XRD ω-2ϑ scans of ZnO-$Sr_3Al_2O_6$ nanocomposite film grown on ZnO(001) substrate before and after dipping in water and drying.

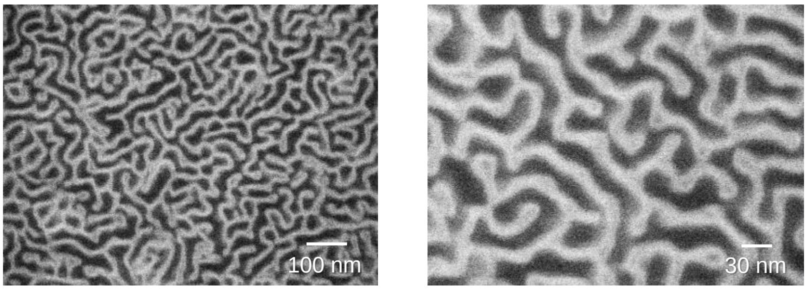

Figure S6. Top-view SEM images of ZnO-$Sr_3Al_2O_6$ nanocomposite film grown on ZnO(001) substrate after dipping in water and drying.

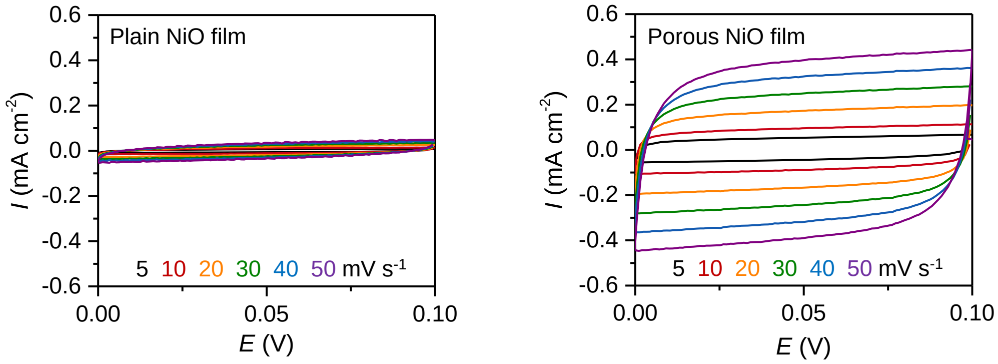

Figure S7. Cyclic voltammograms without faradaic reaction within range from 0 to +0.1 V occurred at different scan rates from 5 to 50 mV s^{-1}.

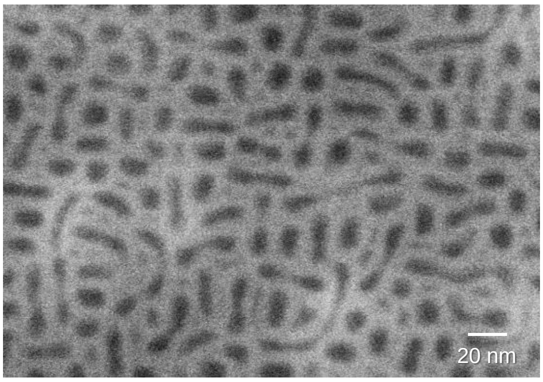

Figure S8. Top-view SEM image of porous NiO film on NiO buffer layer after 100 cycles in the electrochemical test.
